# The Effects of Supplementary Mulberry Leaf (*Morus alba*) Extracts on the Trace Element Status (Fe, Zn and Cu) in Relation to Diabetes Management and Antioxidant Indices in Diabetic Rats

**DOI:** 10.1007/s12011-016-0696-1

**Published:** 2016-04-13

**Authors:** Ewelina Król, Magdalena Jeszka-Skowron, Zbigniew Krejpcio, Ewa Flaczyk, Rafał W. Wójciak

**Affiliations:** 1 Department of Human Nutrition and Hygiene, Poznań University of Life Sciences, ul. Wojska Polskiego 31, 60-624 Poznań, Poland; 2 Department of Chemistry and Technical Electrochemistry, Poznań University of Technology, ul. Berdychowo 4, 60-965 Poznań, Poland; 3 Department of Food Service and Catering, Poznań University of Life Sciences, ul. Wojska Polskiego 31, 60-624 Poznań, Poland; 4 Department of Clinical Psychology, Poznań University of Medical Sciences, Poznań, Poland

**Keywords:** Mulberry leaf extracts, Diabetes, Trace elements, Rats

## Abstract

Mulberry leaves (*Morus alba*) have been used in folk medicine to mitigate symptoms of diabetes. The mulberry plant contains phenolic compounds that are able to decrease blood glucose concentration. Since various phenolics have antioxidant and metal binding properties, they can be used to alleviate oxidative stress and chelate trace elements involved in redox reactions. The aim of this study was to evaluate the effects of dietary supplementation with mulberry leaf extracts (acetone–water (AE) and ethanol–water (EE)) on the trace element status (Fe, Zn and Cu) in relation to diabetes management and antioxidant indices in high-fat diet-fed/STZ diabetic rats. The experiment was performed on 38 male Wistar rats with diabetes (induced by high-fat diet (HF) and streptozotocin injection) or the control fed with AIN-93M or high-fat diet. As a result, five experimental groups were used: (1) a healthy control group fed with AIN-93M; (2) an HF control group; (3) a diabetic HF group; (4) a diabetic HF + AE group (6 g/kg diet); (5) a diabetic HF + EE group (6 g/kg diet). The rats were fed with appropriate diets for 4 weeks. The content of trace elements (Fe, Zn and Cu) in the serum and tissues was measured by means of atomic absorption spectrometry (AAS). Biochemical analyses (glucose, TBARS, FRAP) were performed on the blood serum. It was shown that the AE decreased hepatic and renal Fe stores, while the EE increased hepatic Cu levels in diabetic rats and confirmed their ability to regulate the Fe and Cu status in diabetes. The results confirmed a significant hypoglycaemic and antioxidant potential of both mulberry leaf extracts in diabetic rats.

## Introduction

Diabetes mellitus is a chronic disease in which the body does not produce enough insulin to function properly (type 1) or body cells do not react to insulin (insulin resistance) (type 2). Insulin resistance, defined as impaired responsiveness of the body to insulin, is a prediabetic stage associated with obesity, leading to type 2 diabetes [1]. It is predicted that by the year 2030, over 366 million people worldwide will have been afflicted by type 2 diabetes [2].

Oxidative stress is one of major risk factors in diabetes progression. Lifestyle factors, such as obesity and unhealthy eating habits, as well as increased age disturb redox balance in the body and influence insulin sensitivity [3]. Hyperglycaemia exacerbates oxidative stress and leads to the development of microvascular (e.g. retinopathy, nephropathy, neuropathy) and macrovascular complications (e.g. cardiovascular disease) [4]. Complications of diabetes do not only significantly deteriorate diabetic patients’ health but also increase the costs of healthcare [5].

Therefore, the main therapeutic aim in diabetes is to maintain normal blood glucose levels. In the treatment of type 2 diabetes, depending on the cause and existing metabolic disorders, insulin, insulin secretagogues or sensitizers are used alone or in combination. Another way to diminish blood glucose level is to lower carbohydrate absorption in the gut by using alpha-glucosidase inhibitors [6].

Therefore, there have been increasing efforts in search of natural and synthetic bioactive compounds which can improve insulin action and lower blood glucose levels. One of well-known natural sources of antidiabetic agents is the mulberry plant (*Morus alba L.*). Mulberry leaves and their extracts have been used in folk medicine due to their therapeutic properties, particularly: anti-inflammatory, antidiabetic and diuretic properties [7, 8].

The plant’s leaves contain a variety of nutrients and non-nutrients, including albumins (11.1 %), globulins (9.7 %), prolamins (44.1 %), glutelins (8.5 %) and structural proteins (26.6 %); carbohydrates and fibre [9] and vitamins B (B_1_, B_2_, B_6_, folic acid, PP), C, D, E, β-carotene and xantophylls [9–11]. Besides a number of bioactive phytochemicals, such as flavonoids, steroids, triterpenes, quercetin-3-O-(6-malonyl)-b-d-glucopyranoside, kaempferol-3-O-(6-malonyl)glucoside and quercetin-3-O-glucoside, chlorogenic acids and caffeic acid and 1-deoxynojirimycin (DNJ), an inhibitor of α-glucosidase, have been identified [12, 13]. Andallu et al. [14] found that oral administration of mulberry leaf powder lowered blood glucose, triglycerides, VLDL cholesterol and LDL cholesterol and fatty acids in patients with type 2 diabetes. Also, mulberry leaf ethanol–water extract (included in confections) effectively decreased the postprandial blood glucose and insulin concentration in healthy subjects [15]. Asai et al. [16] showed that long-term ingestion of mulberry leaf extract enriched with DNJ improved postprandial glycaemic control in individuals with impaired glucose metabolism. Hamdy [17] reported that the water extract of mulberry leaves lowers glucose levels in rats with induced diabetes mellitus type 2, regulates the level of oxidative stress and increases the activity of hexokinase, glycogen synthesis and reduces the formation of glucose-6-phosphate in the liver of animals. The mechanisms of the antidiabetic effects of mulberry leaves seem to be multidirectional. The best-known iminosugar, 1-deoxynojirimycin (DNJ), inhibits α-amilase and galactosidase [18], while polysaccharides are competitors of α-glucosidase. Moreover, mulberry leaves contain phenolics with strong antioxidant potential that can mitigate oxidative stress and related complications in diabetes [19, 20].

Polyphenols are known to have a significant affinity to chelate metal ions (e.g. Fe, Cu), and thus, they affect in vivo mineral absorption. Lesjak et al. [21] reported that quercetin can influence intestinal Fe absorption through chelation of Fe ions by 3-hydroxyl groups. High dosages of tannic acid were reported to reduce Fe absorption, but not the absorption of Zn, Cu and Mn in rats [22].

Many studies reported disturbed metabolism of minerals (Fe, Zn and Cu) both in diabetic animals [23–25] and humans [26, 27]. In particular, excessive accumulation of Fe and Cu in the liver and other tissues exacerbates oxidative stress responsible for diabetic complications.

It is hypothesised that when phenolics present in mulberry leaves are administered in diabetes, they could affect the trace mineral status (Fe, Cu), thus attenuating the negative consequence of their tissular excess, which is responsible for oxidative stress and further complications.

The aim of this study is to evaluate the effects of supplementary mulberry leaf extracts (acetone–water and ethanol–water, AE and EE) on diabetes management indices and antioxidant status in relation to the Fe, Zn and Cu status in a combined dietary and pharmacologically induced (STZ) model of diabetes in rats. An excessive amount of fat in the diet is one of factors contributing to insulin resistance. Therefore, a group of rats fed with a high-fat diet was also formed to show biochemical and mineral changes associated with this syndrome.

## Material and Methods

### Preparation of Mulberry Leaf Extracts

Mulberry (*Morus alba L*. Polish variety: wielkolistna zolwinska) leaves were collected from the experimental plant farm of the Institute of Natural Fibres and Medicinal Plants, Poznań, Poland. The leaves (50 kg) were dried in a convection dryer (Rational CCC 61/02, Germany) at 60 °C for 6 h and powdered (0.8–0.08 mm). Optimal solvents and extraction temperatures were chosen according to those applied in our previous study [28]. The optimal extraction conditions were: (1) 65 % acetone/water (*v*/*v*) at 54 °C, repeated three times, and (2) 65 % ethanol/water (*v*/*v*) at 63 °C repeated three times. Combined extracts from each process were filtered using Whatmann filter paper No. 1:11 μm (Whatmann, Bedford, MA, USA) and air-dried. After the evaporation of the solvents, the mulberry leaf extracts obtained, namely the acetone extract (AE) and ethanol extract (EE), were freeze-dried (Christ Alpha 1–4, LSC, Germany).

### Animal Study

Thirty eight 8-week-old male Wistar rats weighing between 214 and 301 g were purchased from the Poznań University of Medical Sciences, ul. Dojazd 30, Poznań, Poland. The rats were kept at the Animal House at the Poznań University of Life Sciences at a temperature of 22 ± 2 °C, humidity of 55 ± 5 % and a 12/12-h light/dark cycle. During the acclimation period, the rats had free access to a standard rat diet (Labofeed B, Andrzej Morawski Feed Production Plant, Kcynia, Poland) and tap water. Then, the animals were randomly allocated using a random number generator at a 1:5 ratio to the standard of the control group fed with the AIN-93M diet (7 % fat, 12.5 % energy, *n* = 6) and the high-fat diet group (HF, *n* = 32) fed with the high-fat diet (25 % fat, 45 % energy, *n* = 32) for 4 weeks. The AIN-93 M and HF diets were prepared by modifying the AIN-93M diet (Table 1) [29]. The diets were prepared weekly and stored in sealed containers at 4 ± 1 °C. The animals had free access to feed and tap water. The food intake was monitored daily, while the body mass gain was monitored weekly. After 4 weeks, the rats from the HF group were randomly divided to four experimental groups (*n* = 8). Diabetes (hyperglycaemia) was induced in three HF groups by a single intraperitoneal injection of STZ (35 mg/kg body mass), freshly dissolved in 0.1 M-citrate buffer (pH 4.4), while one group from the HF diet as well as control group were injected citrate buffer alone in the same manner. The presence of diabetes (DB) in the rats was confirmed by measuring fasting blood glucose concentration withdrawn from the tail tip with a glucometer (Accu-Check®, Roche Diagnostics, Warsaw, Poland) after 48 h. At the second stage of the experiment, the DB rats were subjected to appropriate treatment with mulberry leaf extracts (AE and EE). As a result, the experiment consisted of five groups, as follows: non-DB/control (healthy, AIN-93 M diet); non-DB/no treatment (HF diet); DB/no treatment (HF diet); DB/treatment with AE (6 g/kg HF diet); DB/treatment with EE (6 mg/kg HF diet).

**Table 1:** The composition of experimental diets

*Ingredient* (*g*/*kg diet*)	*Control* (*C*) (*AIN-93M*)	*High fat diets* (*HF*)		
		*HF*	*HF* + *AE*	*HF* + *EE*
Casein	140.0	140.0	140.0	140.0
Lard	0.0	150.0	150.0	150.0
Sunflower oil	70.0	100.0	100.0	100.0
Wheat starch	592.0	412.0	406.0	406.0
Sucrose	100.0	100.0	100.0	100.0
Potato starch	50.0	50.0	50.0	50.0
Mineral mixture^a^	35.0	35.0	35.0	35.0
Vitamin mixture^b^	10.0	10.0	10.0	10.0
L-cystine	3.0	3.0	3.0	3.0
Acetone extract (AE)	0.0	0.0	6.0	0.0
Ethanol extract (EE)	0.0	0.0	0.0	6.0

^a^Mineral mixture was prepared according to AIN-93 M recommendation [29]

^b^Vitamin mixture was prepared according to AIN-93 M recommendation [29]

The rats were fed with appropriate diets for 4 weeks, and then after 16 h of fasting, they were anaesthetised with an intraperitoneal thiopental injection (40 mg/kg b.w.) and dissected to collect blood from the aorta, coagulated at room temperature for 20 min, and centrifuged at 4000 rpm. Serum samples were stored at −70 °C for biochemical assays. Inner organs (liver, kidneys, spleen) were removed, washed in saline, weighed and stored at −20 °C until analysis.

All the experimental procedures were approved by the Animal Bioethics Committee of Poznań, Poland (No. 55/2009).

### Determination of Nutritional and Biochemical Indices

The daily intakes of polyphenols and flavonoids were calculated according to the daily feed intake and the concentration of these compounds in acetone and ethanol extracts determined in the previous study [30].

Fasting blood glucose, serum insulin, ferric reducing ability of plasma (FRAP) and tiobarbituric acid reactive substances (TBARS) were measured as previously described [30].

### *Metal Determination in Rats*’ *Serum and Organs*

Serum samples were diluted twice using Triton-X100 solution. Appropriate tissues were weighed and digested in 65 % (*w*/*w*) spectra pure HNO_3_ (Merck) in the Microwave Digestion System (MARS 5, CEM). The content of Fe, Zn and Cu in the mineral solutions was measured with the flame-AAS method (AAS-3 spectrometer, Carl-Zeiss, with BC, Germany). The accuracy of quantitative determinations of minerals was assured by simultaneous analysis of three certified reference materials (Virginia Tobacco leaves, Poland; Pig Kidney BCR® No. 185, Brussels; serum HUMASY CONTROL 2, Randox, UK). The recovery rate of Fe, Zn and Cu was: 94–106 %, 91–98 %, and 96–102 %, respectively.

### Statistical Analysis

All measurements were performed in triplicate, and the results were expressed as arithmetic mean ± standard error of mean (SEM). Statistical differences in the means between the experimental groups were assessed using one-way analysis of variance (ANOVA) and Tukey’s post hoc test. The means were considered statistically different at *p* < 0.05. All statistical analyses were performed using Statistica 10.0 software (Statsoft Corp., USA).

## Results

There were differences found in the mineral composition of extracts. Fe and Cu contents were higher in the EE, while the AE was characterised by higher levels of Zn (Table 2).

**Table 2:** The content of minerals in *Morus alba* leaves extracts (mg/100 g dry matter)

Item	*Morus alba* leave extracts	
	AE (65 % acetone–water)	EE (65 % ethanol–water)
Fe	1.163 ± 0.008a	2.783 ± 0.040b
Zn	7.439 ± 0.338b	4.500 ± 0.088a
Cu	0.344 ± 0.001a	1.249 ± 0.013b

Data are mean ± SEM; means in a row with different letters differ significantly (*p* < 0.05)

**Fig. 1:**
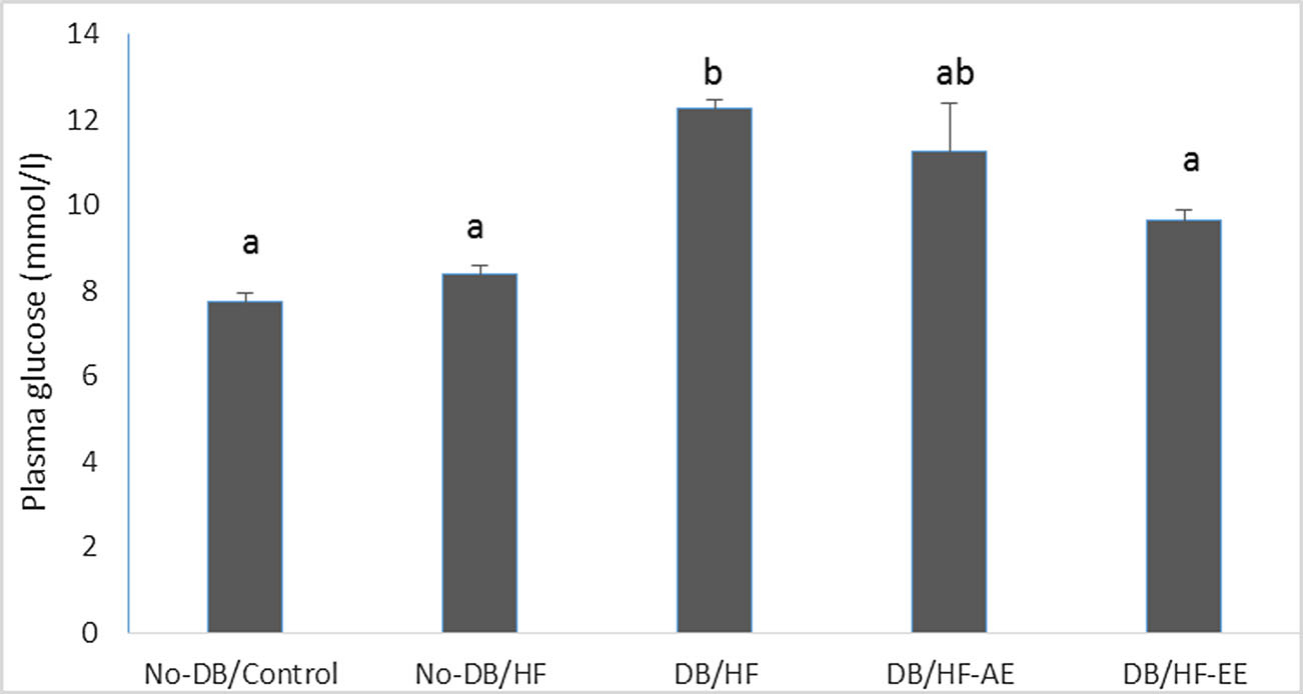
Plasma glucose concentration at the end of the experiment. Data are mean ± SEM; bars with different letters differ significantly (*p* < 0.05).

Table 3 and Fig. 1 show the effects of feeding the rats with the high-fat diet (HF), STZ injection and supplementary AE and EE on nutritional and blood biochemical indices. In our previous article [26], the chemical compositions of mulberry leaf (*Morus alba*) acetone–water extract (AE) and ethanol–water extract (EE) were characterised. The EE derived from mulberry leaves contained higher amounts of bioactive compounds than did the AE, i.e. total phenolics, total flavonoids, chlorogenic acid, caffeic acid, vanillic acid quercetin and rutin, isoquercetin, kaempherol, astragalin. This resulted in higher total polyphenols and flavonoids intake in both experimental groups (Table 3). Additionally, due to the higher content of these compounds in the EE, the intakes of chlorogenic acid, rutin and quercetin 3-(6-malonylglucoside) were higher. However, neither extract affected the diet intake significantly.

**Table 3:** Nutritional and blood biochemical indices in rats

Index	Experimental group				
	No-DB/control (C)	No-DB/HF-no treatment	DB/HF-no-treatment	DB/HF-AE	DB/HF + EE
Diet intake (g dm/day)	20.20 ± 0.45b	16.79 ± 0.59a	16.96 ± 0.60a	17.85 ± 0.35a	16.84 ± 6.87a
Dietary total phenolics intake (mg GAE/kg bw/day)	−	−	−	24.83 ± 0.19a	31.57 ± 0.19b
Dietary total flavonoids intake (mg QUE/kg bw/day)	−	−	−	10.63 ± 0.23a	13.05 ± 0.22b
Chlorogenic acid intake (mg/kg bw/day)	−	−	−	1.76 ± 0.08a	3.76 ± 0.03b
Quercetin 3-(6-malonylglucoside) intake (mg/kg bw/day)	−	−	−	2.45 ± 0.03a	3.38 ± 0.02b
Rutin intake (mg/kg bw/day)	−	−	−	1.22 ± 0.01a	1.68 ± 0.01b
Serum Fe (μmol/L)	30.95 ± 3.50	37.86 ± 3.51	35.71 ± 3.66	35.88 ± 5.24	26.13 ± 4.28
Serum Zn (μmol/L)	20.00 ± 0.76bc	16.62 ± 0.62ab	15.94 ± 0.88a	17.15 ± 0.58ab	16.11 ± 0.50ab
Serum Cu (mmol/L)	16.72 ± 0.47	14.69 ± 0.48	14.97 ± 1.42	14.88 ± 0.53	15.52 ± 0.52
Serum Zn/Cu (molar ratio)	1.20 ± 0.05	1.14 ± 0.07	1.11 ± 0.05	1.16 ± 0.04	1.05 ± 0.06
Serum TBARS (μmol TMP/L)	8.71 ± 0.28 a	17.91 ± 4.48ab	22.52 ± 4.83b	11.39 ± 1.28a	11.12 ± 0.89a
Serum FRAP (μmol FeSO_4_/L)	383.93 ± 13.75c	320.05 ± 20.92ab	323.64 ± 7.39ab	307.64 ± 14.41a	350.34 ± 14.47abc
Serum insulin (pmol/L)	478.51 ± 32.67b	369.05 ± 20.29b	130.54 ± 4.78a	479.52 ± 88.54ab	374.10 ± 36.28b

Data are mean ± SEM; means in a row with different letters differ significantly (*p* < 0.05)

*TBARS* thiobarbituric acid reactive substances, *TMP* 1,1,3,3-tetramethoxypropane, *DNJ* 1-deoxynojirimycin, *FRAP* ferric reducing ability of plasma

In comparison with the AIN-93M standard diet (7 % fat, 12 % of energy), the high-fat diet (HF, 25 % *w*/*w*, 45 % of energy) significantly decreased the diet intake (by 23 %). On the other hand, the HF diet significantly increased the liver Fe content (by 60 %) and decreased the kidney Cu content (by 25 %).

The intraperitoneal STZ injection induced hyperglycaemia (diabetes) in rats by damaging the pancreatic β-cell function, resulting in significantly decreased serum insulin concentration (by 73 %), increased plasma glucose level (by 46 %) as well as decreased serum Zn concentration (by 20 %). On the other hand, hyperglycaemia increased the lipid peroxidation process, which was evidenced by an elevated serum TBARS value (by 153 %). Furthermore, the hyperglycaemic (DB) rats had significantly higher renal Fe and Cu contents (by 43 and 52 %) than did non-diabetic (non-DB) rats.

Mulberry leaf extracts (AE and EE, both 6 g/kg diet, ca. 0.5 g/kg body mass/day) given to the hyperglycaemic (DB) rats attenuated some nutritional and blood biochemical indices to various extents, depending on the type of extract. In general, both extracts showed appreciable hypoglycaemic effects evidenced with decreased blood glucose and elevated insulin concentration (Table 3, Fig. 1). However, the EE displayed a slightly stronger hypoglycaemic effect (containing higher amount of total phenolics and flavonoids) than the AE did. Also, both extracts improved antioxidant capacity in the rat organism, which was clearly demonstrated by a decreased serum TBARS value (by 50 %). FRAP, serum Fe, Zn and Cu as well as serum Zn/Cu molar ratio was comparable in all diabetic groups.

Furthermore, supplementary mulberry leaf extracts had impact on Fe and Cu, but not on Zn levels in the diabetic rats’ internal organs. In particular, the AE appeared to have a stronger effect than the EE, as it significantly decreased liver and kidney Fe concentration (by 25 and 22 %), while the EE increased the liver Cu content (by 22 %). It was associated with a decreased liver Zn/Cu molar ratio (by 20 %) (Table 4).

**Table 4:** The content of microelements (Fe, Zn and Cu; in μg/g dry matter) in tissues of experimental rats

Mineral content (μg/g dm)		Experimental group				
		No-DB/control (C)	No-DB/HF-no treatment	DB/HF-no-treatment	DB/HF-AE	DB/HF + EE
Fe	Liver	266.31 ± 19.10a	424.64 ± 24.72b	415.14 ± 23.39b	313.63 ± 24.51a	338.46 ± 15.45ab
	Kidneys	298.31 ± 10.41ab	257.84 ± 3.70a	369.67 ± 16.16b	288.67 ± 13.12a	304.24 ± 11.65ab
	Spleen	3705.4 ± 214.65	3697.5 ± 108.91	3612.8 ± 469.19	2661.8 ± 279.29	3368.5 ± 286.15
Zn	Liver	96.79 ± 3.07	90.87 ± 3.81	97.07 ± 1.77	95.25 ± 2.44	107.07 ± 3.21
	Kidneys	99.78 ± 2.22	86.47 ± 3.97	100.43 ± 3.20	103.04 ± 5.24	101.73 ± 5.01
	Spleen	80.83 ± 2.63	83.64 ± 2.55	88.26 ± 1.15	83.09 ± 2.56	81.26 ± 1.11
Cu	Liver	18.93 ± 0.78ab	15.73 ± 0.86a	18.28 ± 0.29a	17.75 ± 0.94a	22.35 ± 1.12b
	Kidneys	39.77 ± 2.01b	29.77 ± 2.58a	45.25 ± 5.34b	42.98 ± 2.49b	43.59 ± 4.25b
	Spleen	10.52 ± 0.75	12.64 ± 0.44	17.00 ± 2.80	17.78 ± 2.59	8.88 ± 1.62
Zn/Cu (molar ratio)	Liver	5.07 ± 0.18ab	5.71 ± 0.16 b	5.24 ± 0.12ab	5.32 ± 0.29ab	4.55 ± 0.19a
	Kidneys	2.49 ± 0.08	2.98 ± 0.24	2.56 ± 0.23	2.37 ± 0.23	2.32 ± 0.19
	Spleen	8.02 ± 0.54	6.53 ± 0.30	6.12 ± 0.83	6.60 ± 1.22	10.49 ± 1.40

Data are mean ± SEM; means in a row with different letters differ significantly (*p* < 0.05)

## Discussion

Our previous article [30] showed that the mulberry leaf ethanol–water extract (EE) with a higher level of phenolics–chlorogenic acid and flavonol glucosides was more effective than the acetone–water extract (AE) or dry leaves was in lowering blood glucose, increasing insulin level and markers of antioxidant activity in the STZ-induced non-obese diabetic rat model. In this study, we focused on the effects of supplementary mulberry leaf extracts (EE and AE) on the Fe, Zn and Cu status in relation to hypoglycaemic and antioxidant capacity in diabetic rats.

It is well known that essential trace elements, especially Fe, Zn, Cu and Mn play a key role in various biochemical redox reactions as catalytic centres of various enzymes. Both deficiency and excess of these micronutrients disturb the antioxidant balance, increase free radical formation and oxidative stress in cells and tissues. Hyperglycaemia typical of diabetic states is associated with increased protein glycation, inflammation and oxidative stress due to excessive free radical formation [31]. Many observations showed that the metabolism of some essential elements (Mg, Fe, Zn, Cu) is disturbed in insulin resistance and diabetic states [32]. On the other hand, disturbed Fe metabolism, especially Fe overload, can result in glucose intolerance. Iron is a trace element which produces reactive oxygen species (ROS) participating through the Fenton reaction and ROS may cause oxidative stress and further diabetic complications. Recently, Morita et al. [23] have shown that type 1 diabetes increases renal tubular Fe accumulation and macrophage infiltration through a p21-dependent mechanism and that the chelation of dietary Fe attenuates these responses.

Kim et al. [26] found that Fe overload is associated with insulin resistance in men, but not in women. The results of another study indicate that elevated serum ferritin levels (without evident Fe overload) may affect glucose homeostasis, leading to insulin resistance in conjunction with inflammatory changes (seen as elevated C-reactive protein levels) [33]. Salmonowicz et al. [27] reported that T1DM children had lower plasma levels of Mg and Zn and higher levels of Cu; lower Cu/Zn SOD activity; higher catalase (CAT) activity and lower total antioxidant status (TAS) levels.

Usually, the first step in diabetes progression is insulin resistance, which can be studied on an animal model by feeding it with a high-fat diet. This diet can impair the uptake and distribution of Fe, Zn and Cu. Meli et al. [34] investigated the influence of a high-fat diet on Fe metabolism in rats and found that the activity of Fe regulatory protein-1 (IRP1) increased in a time-dependent manner, resulting in increased transferrin receptor-1 and ferritin expressions in the liver. Another study reported that a high-fat diet increased hepatic Fe stores in rats [35]. The same effect of the high-fat diet on hepatic Fe was observed in this study.

Another microelement involved in glucose metabolism is Zn, which is necessary for insulin production, activation and storage. Zn is a component of plethora of proteins and enzymes, including those involved in the maintenance of antioxidant balance (e.g. metallotionein, Cu/Zn SOD). Soinio et al. [36] found that alterations in Zn metabolism induced by prolonged hyperglycaemia may increase oxidative damage of cells and exacerbate complications in diabetes. The efficacy of regulation of Zn transporters plays a key role in the pathogenesis of diabetes [37]. It was found that SLC30A8 gene encoded Zn transporter 8 is linked with the occurrence of type 1 diabetes [38]. Additionally, in in vitro studies, Zn can induce an increase in glucose transport into cells as well as potentiate insulin-induced glucose transport [39]. Zinc is believed to act through the insulin-signalling pathway and to some extent, it has insulin-mimetic properties.

Copper is an integral component of Cu-containing proteins and enzymes (e.g. ceruloplasmin, Cu-Zn SOD, cytochrome c oxidase), which are involved in electron transfer reactions. Cu–Zn superoxide dismutase and ceruloplasmin play antioxidant functions in the body and are essential to aerobic organisms. It is reported that Cu metabolism may be disturbed in diabetes. However, the results obtained in experimental studies on animals are still inconclusive. For example, in diabetic rats, hepatic and renal Cu levels were increased and cardiac catalase (CAT), glutathione S-transferase (GST), Cu–Zn SOD and MnSOD activities were elevated [24]. Some authors [25] reported that splenic Cu concentration was increased, while Cu levels in the serum, liver, heart and cerebellum were decreased in a rodent model of diabetes. Changes in Zn and Cu levels were also reported in diabetic patients. Elevated serum Cu concentration and Cu/Zn ratio as well as decreased serum Zn levels were noticed by Victorinova et al. [40]. Also, in diabetic patients (both type 1 and 2), glycated haemoglobin levels were positively correlated with serum Cu concentration and Cu/Zn ratio, while they were inversely correlated with serum Zn concentration. However, the mechanisms responsible for changes in Cu and Zn levels in diabetes are not fully known. According to Eaton and Qian [41], chronic hyperglycaemia increases blood protein glycosylation and these glycated forms have higher affinity to transition metals, especially Cu^+2^ ions, resulting in increased absorption and deposition of Cu in critical organs. It is known that imbalance between Cu and Zn in tissues, particularly an excess of Cu in relation to Zn, triggers the formation of free radicals and oxidative stress, which aggravate complications in diabetes. Disrupted homeostasis of Cu or Zn in diabetics is correlated with neuropathy, retinopathy as well as coronary heart disease [36, 41, 42]. Zheng et al. [43] reported that the application of chelating agents that selectively bind Fe and Cu ions could be a useful approach to mitigate complications in diabetes.

There are no publications on the effects of mulberry leaf extracts on the trace element status in diabetes in the available literature. However, some experiments have been conducted to show the effect of a chemically pure form of polyphenols on trace element metabolism. According to Hunyadi et al. [18], chlorogenic acid and rutin are the main components of mulberry leaves responsible for their antidiabetic activity. In the experiment reported by Gao et al. [44], mice receiving rutin by gavage at concentrations of 0.75 and 2.25 g/kg b.m. for 30 days had decreased hepatic Fe, Zn and Cu contents. Similar results were obtained when mice were fed with a diet with rutin or baicalein (1 % diet) for 20 days [45]. According to these authors, flavonoids can bind metal ions in vivo, thus reducing their uptake and storage in internal organs.

In this study, mulberry leaf extracts contain a number of phenolic compounds (e.g. quercetin) that have proven metal chelating affinity [46]. Quercetin, rutin, kaempferol, flavanol and catechin have documented Cu^+2^ and Fe^+3^ chelating properties [47]. Symonowicz and Kolanek [48] reported that metal-flavonoid complexes may have even stronger free radical scavenging potential than flavonoids alone do. The biological activity of plant phenolics depends mainly on their type, composition, dose and in vivo bioavailability [49]. The bioavailability of flavonoids from food matrix depends on the type of compound, degree of release, sugar moieties and emulsification of dietary fats [50].

The intestinal absorption and biological effects of bioactive compounds of mulberry leaves depends on their composition and dose. As far as extracts are concerned, the type of solvent and extraction conditions determines the efficacy and chemical composition of the product. Lee et al. [51] studied the intestinal absorption of antioxidants from mulberry leaves and found that the absorption of compounds from the ethanol extract was higher than from the aqueous one. In this study, the supplementary ethanol–water extract of mulberry leaves (EE) increased the hepatic Cu content (by 22 %) and decreased the hepatic Zn/Cu molar ratio (by 20 %) in diabetic rats, while the acetone–water extract (AE) did not. This may have been caused by three times higher Cu content in the EE than in the AE.

Based on the results obtained in this study, we hypothesised that supplementary mulberry leaf extracts (AE and EE) given to diabetic rats (with disturbed Fe and Cu metabolism) may interact with Fe and Cu ions, reducing their uptake, modifying distribution and storage and/or increasing their renal excretion. This helps to correct Fe and Cu overload and mitigate oxidative events in diabetes.

## Conclusions

This study focused on the effects of mulberry leaf (*Morus alba*) extracts (AE and EE) as rich sources of plant polyphenols on glucose management indices and overall antioxidant status in relation to the trace element (Fe, Zn and Cu) status in diabetic rats. The results confirmed the significant hypoglycaemic and antioxidant potential of both acetone–water and ethanol–water extracts as well as their ability to regulate the Fe and Cu status in diabetes.

It seems that the application of natural plant phenolic compounds to control glucose management and alleviate complications caused by oxidative stress might be a useful approach in the treatment of diabetes. The mechanisms of in vitro polyphenols—trace elements interactions are not fully known and require further investigation.
